# Spanish Teachers’ Perceptions of Their Preparation for Inclusive Education: The Relationship between Age and Years of Teaching Experience

**DOI:** 10.3390/ijerph19095750

**Published:** 2022-05-09

**Authors:** Natalia Triviño-Amigo, Sabina Barrios-Fernandez, Carlos Mañanas-Iglesias, Jorge Carlos-Vivas, María Mendoza-Muñoz, José Carmelo Adsuar, Ángel Acevedo-Duque, Jorge Rojo-Ramos

**Affiliations:** 1 Social Impact and Innovation in Health (InHEALTH) Research Group, Faculty of Sport Sciences, University of Extremadura, 10003 Caceres, Spain; natalia.trivino@alu.uhu.es (N.T.-A.); cmaanasi@alumnos.unex.es (C.M.-I.); jorgerr@unex.es (J.R.-R.); 2 Promoting a Healthy Society Research Group (PHeSO), Faculty of Sport Sciences, University of Extremadura, 10003 Caceres, Spain; jorgecv@unex.es (J.C.-V.); jadssal@unex.es (J.C.A.); 3 Research Group on Physical and Health Literacy and Health-Related Quality of Life (PHYQOL), Faculty of Sport Sciences, University of Extremadura, 10003 Caceres, Spain; mamendozam@unex.es; 4 Departamento de Desporto e Saúde, Escola de Saúde e Desenvolvimento Humano, Universidade de Évora, 7004-516 Évora, Portugal; 5 Public Policy Observatory, Universidad Autónoma de Chile, Providencia 7500912, Chile

**Keywords:** inclusive education, teachers, initial preparation, ongoing preparation, perceptions

## Abstract

Inclusive education (IE) refers to the education of all learners, with or without disabilities, irrespective of their status or origin, who share the same learning spaces. IE is a multidimensional approach based on rights and quality of life paradigms. As teachers are agents of change, they must have the knowledge and competencies to meet this challenge. This study aimed to find potential associations between teachers’ preparation and their age and years of teaching experience. A cross-sectional study with a sample of 1275 teachers working in early childhood, primary and secondary education was performed. They answered three dichotomic questions about their initial and ongoing preparation and The Evaluation of Teacher Preparation for Inclusion (CEFI-R) Questionnaire. The dichotomic questions showed that 26.4% of respondents felt qualified to face the challenges of their students’ diversity. There were significant inverse associations between the CEFI-R Dimension 1 (diversity conception), Dimension 3 (supports), and Dimension 4 (community participation) and the teachers’ age and years of teaching experience. This means that the higher the age or the years of experience, the teachers’ perceived preparation for inclusion is worse, which should encourage us to take measures to improve teachers’ competencies and preparedness.

## 1. Introduction

Inclusive education (IE) refers to the education of all learners, with or without disabilities, irrespective of their status or origin, and involves reinforcing the capacity of the education system to welcome and reach out to all learners [1]. It is a multidimensional approach that accepts diversity based on the paradigms of rights and quality of life [2,3]. Inclusion involves the right to education for all students; thus, IE values focus on partnership, participation, democracy, profit, equal access, quality, equity, and justice [4,5]. Inclusive schools must create an optimal system that meets every student’s needs and must be prepared to cope with the different demands [6]. Furthermore, IE accepts and celebrates cultural, sexual, social, religious, ethnic, or linguistic diversity [7]. Thus, IE pursues the transformation of the educational system so that all students can develop their personalities and learning, providing them with appropriate support and building the foundations for a respectful and sustainable society [2,8].

For more than 40 years, different organizations, such as the United Nations Educational, Scientific and Cultural Organization (UNESCO) (https://www.unesco.org, accessed on 11 February 2022), the European Agency for Special Needs and IE (https://www.european-agency.org, accessed on 11 February 2022), the Center for Studies on IE (http://www.csie.org.uk, accessed on 11 February 2022), Plena Inclusión (https://www.plenainclusion.org, accessed on 11 February 2022) and many others, have been working to promote IE. As a result, documents such as the Warnock Report [9], the Salamanca Statement [10], and the Index for Inclusion [8] have emerged. More recently, the United Nations (UN) created 17 sustainable development goals (SDGs) [11]. Goal 4, “Quality Education”, states that efforts should be focused on creating quality education for all, promoting citizenship and inclusion, and elaborating the Incheon Statement, providing a framework to promote quality education [12]. According to UNESCO [13], many countries are currently working toward implementing IE; although it is a considerable challenge for educational systems, there are still barriers that prevent its full implementation [14]. IE is sometimes considered a reductionist approach toward incorporating students with disabilities in regular education contexts [15]. However, there is a growing understanding that it should be a framework to develop and deliver educational policies and practices that support all learners [7,15].

The evolution from exclusion to segregation in Spain and from integration to an inclusive stance has been influenced by the international context, as mentioned above [16]. Students with disabilities were entirely excluded until the Public Education Act of 1857, in which a small amount of progress was made toward segregation. In the General Law on Education and Financing of Educational Reform (1970), the concept of special education was established, and in 1985, the Royal Decree 334 on the organization of special education was approved, proposing the integration of students with disabilities into the mainstream education system. In 2006, the Organic Law on Education (LOE) committed to inclusion, regardless of students’ personal and social conditions. In 2013, the Law on Education for the Improvement of the Quality of Education (LOMCE) introduced a few significant changes to IE [16]. Nowadays, the Organic Law 3/2020, which amends the Organic Law 2/2006 on Education (LOMLOE) [17], is the current legislation on educational system organization and dictates the educational response regarding students with diversity, complemented by the decrees to be developed by each of the Spanish regions. On the one hand, the Spanish educational system structure comprises several levels: early childhood education has two stages, one being between 0 and 3 years (non-compulsory); the second one, primary, is from 3 to 6 years. Primary education is compulsory and includes six grades intended for ages 6 to 12; secondary education is divided into mandatory secondary education between 12 and 16 years. Students can choose between professional training or the baccalaureate to prepare them for university. On the other hand, and in terms of diversity, LOMLOE states that an individualized response must always be given to each student, considering the families’ opinions. It also establishes that the most inclusive pathway must be chosen for each student. Therefore, since its promulgation, the educational system has had ten years for special education centres to be transformed into resource centres for IE support and promotion [17,18].

Teachers play a crucial role in this transformation within schools. Therefore, some factors must be considered, including their initial and ongoing preparation, professional experience, perceived self-efficacy, beliefs, the types of students with disabilities with whom they will work, and the available educational resources to deal with the diversity of their students [19]. A growing debate is being held on whether university curricula provide future teachers with adequate knowledge and competencies for IE promotion [20,21], as this initial preparation will guide the identity and development of the teachers [22]. Some proposals include incorporating IE, poverty issues, and climate change-related content, calling for a more sustainable and comprehensive education curriculum [23]. Moreover, more attention should be paid to ongoing education, as only some teachers attend these courses of their own free will [24]. Teachers’ preparations must also incorporate competencies to face the challenges related to IE: if teachers feel highly effective, their capacity for innovation and their determination to do their work correctly will be enhanced [25,26]. Thus, teachers’ self-efficacy or personal belief in their abilities to address challenging situations must be considered [26,27]. Another aspect is the lack of specialized resources for both students and teachers during the educational process [19,28,29]; however, educational agents express this as a significant barrier preventing students with disabilities from being included. Contact with students with disabilities is another aspect to consider: if future teachers and teachers do not have direct contact with students with disabilities, their conceptions and attitudes may not match reality, so their initial and ongoing preparation must help to overcome possible incorrect attitudes and beliefs [30,31,32,33,34]. Furthermore, IE requires teachers to assume new roles, including conducting diagnostics on educational policies, practices, and cultures, determining whether they are inclusive or need to be transformed [8,12,35].

Teachers undergo a series of phases throughout their careers, depending on the length of time they have been teaching, which influences their ability to develop educational practices [36,37]. Huberman’s model [38] postulates five stages, with the second stage (3–7 years of teaching) being the one in which work activities stabilize. Although other classifications exist [39], they all emphasize that the first phases are related to “survival” and knowing and assuming their new role, trying to accommodate their vision of education and their situation. However, it should not be forgotten that teachers face many stressors [40], including job instability, which often makes it challenging to plan their personal and professional future, influencing their self-realization and affecting their professional performance [41]. Some studies have shown that disability conception tends to be more positive in younger teachers (20–30 years old) compared to older groups (40–50 years old) [42].

For all the above reasons, this study aims to analyze the teachers’ self-perceptions of their preparation for IE at different educational stages (infant, primary and secondary) in Spain, searching for differences according to their ages and years of teaching experience.

## 2. Materials and Methods

### 2.1. Study Design and Ethical Concerns

This manuscript presents a descriptive cross-sectional study design since we aimed to examine data from a specific population at one point in time. The study was conducted according to the Declaration of Helsinki guidelines and was approved by the Bioethics and Biosafety Committee of the University of Extremadura (protocol code: 186/2021).

### 2.2. Procedure

Firstly, an email was sent to the public schools in Extremadura, the contact details of which appeared in the Department of Education and Employment of the Regional Government of Extremadura database (accessed on 10 December 2020). The email included basic information about the study, an informed consent form, and a link to Google Forms (Google, Mountain View, CA, USA) for a sociodemographic survey that was created ad hoc, with three dichotomous questions about their perception of their initial and ongoing preparation to address IE, and the teacher preparation for inclusion evaluation questionnaire (CEFI-R) [43,44]. It was estimated that the respondents’ participation in the study would require about 10–15 min.

### 2.3. Participants 

Finally, 1275 teachers who met the eligibility criteria participated in the study: (1) being a teacher in a public pre-school, primary, or secondary school, and (2) providing signed informed consent to the researchers. Thus, a non-probability sampling method based on convenience sampling was used [45], as recruitment was carried out by emails to schools and teachers who wished to participate.

### 2.4. Instruments

A sociodemographic survey was created ad hoc for this project, including sex, age, the educational stage the participant is teaching, and years of teaching experience.

Three dichotomous questions about their initial and ongoing preparation are posed. (1) Do you think that you were adequately prepared by your initial preparation to respond to the diversity of your students’ needs? (2) Has ongoing preparation helped you to respond to the diversity of your students’ needs? (3) Would you be willing to attend courses on IE? These basic questions are intended further to compare the different educational actors and educational stages.

The Evaluation of Teacher Preparation for Inclusion Questionnaire (CEFI-R) [43] was initially formed of 19 items, grouped into four dimensions (see Table 1): Dimension (1)—diversity conception (5 items); Dimension (2)—methodology (5 items); Dimension (3)—supports (4 items); and Dimension (4)—community participation (5 items). Dimension (1) focuses on the participant’s beliefs regarding the concept of diversity; Dimension (2) relates to the design and development of an inclusive curriculum; Dimension (3) refers to the roles of teachers with their students; while Dimension (4) measures the participation of educational agents in educational practice. Responses were given according to a Likert scale, from 1 (strongly disagree) to 4 (strongly agree). The original authors reported a global Cronbach’s alpha value of 0.79, with each dimension above 0.70 considered good [46]. Its original version in Spanish can be found in Appendix A. Subsequently, a study was carried out on the psychometric properties of this instrument through confirmatory factor analysis, finding a model with good adjustment and goodness-of-fit indicators [44]. 

### 2.5. Statistical Analysis

The statistical package for social sciences (SPSS), version 23 for Mac (IBM SPSS, Chicago, IL, USA), was used to perform the analyses. First, the Kolmogorov–Smirnov test was conducted to determine if the data followed a normal distribution. As this assumption was not met (Kolmogorov–Smirnov = 0.300; *p* ≤ 0.001), non-parametric tests were chosen. Spearman’s Rho was performed to explore the association between the CEFI-R dimensions and the teachers’ ages and years of experience. Correlation coefficients were interpreted following these criteria [35]: 0.00, no correlation; 0.01 to 0.10, weak; 0.11 to 0.50, medium; 0.51 to 0.75, considerable; 0.76 to 0.90, very strong; and 0.91 to 1.00, perfect. Cronbach’s alpha coefficient was used to calculate the reliability of every dimension from the CEFI-R questionnaire [47], considering 0.60–0.70 to be acceptable and 0.70–0.90 to be excellent [46]. 

The variable age was categorized into four age groups: below 30 years, between 31 and 40 years, between 41 and 50 years, and over 50 years, while the years of teaching experience were presented as a continuous variable.

## 3. Results

Table 2 presents the participants’ sociodemographic characterization, composed of 1275 teachers. The mean of the years of teaching experience was 15.01, with a standard deviation of 10.18. A non-probability sampling method based on convenience sampling was used [45].

Table 3 summarizes the answers to the three dichotomous questions about their initial and ongoing teaching preparation.

Table 4 shows the correlation coefficients between the CEFI-R questionnaire dimensions and the years of teaching experience. The dimensions (1) diversity conception, (3) supports, and (4) community participation were significant and were inversely correlated with the years of teaching experience.

Table 5 shows the correlation coefficients between the CEFI-R questionnaire dimensions and teachers’ age, showing an inverse correlation in the same dimensions above: (1) diversity conception, (3) supports, and (4) community participation.

Finally, Table 6 presents the reliability results for every CEFI-R dimension using Cronbach’s alpha. Values are considered good for Dimension (1)—diversity conception and (3) supports, and excellent for Dimensions (2) methodology, and (4) community participation [46].

## 4. Discussion

### 4.1. Main Findings

This research aimed to explore potential associations between Spanish teachers’ perception of their preparation for IE and the teachers’ age and years of teaching experience. On the one hand, regarding the results of the three dichotomous questions, only 26.4% felt qualified to face the challenges of their students’ diversity. However, 77.3% indicated that ongoing preparation helped handle these tasks, while 89.9% stated they are or would be willing to attend EI courses. On the other hand, the CEFI-R results, composed of 19 items and defined by four dimensions [43,44] ((1) diversity conception; (2) methodology; (3) supports; and (4) community participation), showed that older teachers and teachers with more years of teaching experience felt they were less prepared for IE. Moreover, significant associations were found in this study between the dimensions of (1) diversity conception, (3) supports, and (4) community participation, along with teachers’ ages and years of teaching experience.

Although the literature is considerable on the associations between teachers’ age and experience and their attitudes toward disability and IE [42,48,49,50,51,52,53,54,55], studies on teachers’ perceptions of their preparedness to face challenges in IE are limited. One study indicated that 37.5% of early childhood and primary school teachers, and 25% in secondary education, considered themselves unprepared to address IE demands [56], which is in line with our results. Another study about teachers’ preparation in open classrooms [57] found that 61% felt that their initial preparation was of poor quality, either not at all adequate (16.6%) or not very adequate (44.4%). Only 39% considered it quite adequate (31.6%) or very adequate (7.4%). Statistically significant differences were found in the length of teaching experience at the school, which is also following our results. Lastly, another study involving special education teachers found that novice teachers showed lower confidence levels in their preparation. Thus, concerning their years of teaching experience, those with more than ten years of experience showed greater confidence in their skills than those who had worked for five years or less [58]. Therefore, both the studies mentioned above and our results suggest the need to focus especially on novice teachers and those with more years of teaching experience, as they may feel that they are not well prepared to deal with the diversity that IE implies.

Concerning ongoing preparation, one study with special education teachers showed a significant association between teachers who had taken IE university courses and their preparedness to demonstrate seven of the eight skills associated with IE on a survey (individualized instructions for students with significant disabilities, pacing instructions, providing accommodations and support, adapting general education content standards, making appropriate and timely provision to respond to questions using different communication modes, planning for the implementation of goals, using data collection procedures to monitor the students’ progress, and individualizing the evaluation criteria for students with disabilities) [59]. A study performed in Spain using the CEFI-R showed that in terms of age, those teachers under 30 years of age scored significantly lower on the educational policy dimension, while those over 50 years of age scored significantly higher on leadership and significantly lower on resources and support; regarding their years of teaching experience, significant associations were found between more years of experience and education policy and administration, which would be consistent with a more profound knowledge of the functioning and management of the education system. However, a negative correlation was found between the resources and support dimensions, which could reveal a critical opinion regarding the lack of resources in the classroom [60].

### 4.2. Practical Implications

Teachers must display diverse competencies to meet the guidelines of inclusive education. The European Agency for Development in Special Needs Education [35,61] proposes four competencies: valuing diversity, supporting and having high expectations of students, working in a team, and developing professional and personal dimensions. One identified challenge, and why the revision of curricula in initial education is advocated, is that teacher preparation is often mainly oriented toward acquiring knowledge while neglecting attitudes and values [62]. However, to achieve these competencies, teachers’ initial preparation should include, among other measures, practical preparation with experienced teachers in inclusive settings [35,63]. This will lead to higher self-efficacy, influencing teachers’ attitudes toward disability [64,65]. Therefore, exploring teachers’ perceptions of their preparation is relevant, as it impacts their self-efficacy and attitudes and, therefore, their competencies and daily educational practice. Furthermore, to comprehensively address this transformation, ongoing preparation, including course offerings and voluntary attendance at courses, should also be reviewed as being essential, including positive effects on attitudes towards IE [66].

Teachers face a multitude of challenges, including the requirement to comply with high-quality standards, high classroom ratios, difficulties in relationships with families, lack of resources, uncertainty about their future, loss of social prestige, student demotivation and lack of discipline, and the challenge that concerns us in this study, insufficient pedagogical preparation [40,41,67]. Thus, caring for their well-being and quality of life is imperative to carry out their work correctly, including IE tasks, and because it is their right to have appropriate working conditions [68].

In short, according to our results the practical applications of this study are (1) the need to establish the required support for novice teachers, as they are adjusting to their job during the first few years, as well as the need to help and support older and more experienced teachers, as they perceive their preparation to be insufficient to meet their students’ diversity of needs and promote IE; (2) to highlight a profound issue regarding the content and competencies that should be taught during initial teacher preparation, as teachers do not feel confident in facing the demands of their students with disabilities; (3) to support the need for teachers’ ongoing preparation to help them address the educational needs of all their students; (4) to support the need to establish a system for monitoring the preparation of teachers, as it is currently voluntary; (5) with the actions described above, the educational system will help to meet the Declaration of the Rights of the Child [69], the Convention on the Rights of Persons with Disabilities [70], and Goal 4 of the UN’s sustainable development goals [11,12] because the development of educational practices and policies to promote IE will soon be required.

### 4.3. Limitations and Future Lines

The limitations of this study include the following: (1) all participants were teachers from the region of Extremadura, so there might be socio-cultural and legislative differences with other Spanish regions since education is a devolved matter in each autonomous community. (2) Convenience, non-probabilistic, and non-random sampling was used possibly, the sample may not be representative. (3) The study’s design does not allow cause-effect relationships to be established. (4) Online questionnaires were used because of their advantages, although disadvantages of sampling and response rates exist [71]. In the future, it is planned to extend this study to other regions, carrying out a multi-centre study to test whether legislative and cultural differences impact teachers’ perceptions.

## 5. Conclusions

The three dichotomous questions results showed that only 26.4% of teachers felt competent to address their students’ diversity. In contrast, 77.3% indicated that ongoing preparation was helpful, and 89.9% stated they would be willing to attend IE courses. The CEFI-R results showed significant inverse associations between the dimensions (1) diversity conception, (3) supports and (4) community participation, as well as between teachers’ age and the number of years of teaching experience. In other words, the older the age or, the more years of experience of teachers, the worse their perception of their inclusion preparation is.

Therefore, education administrations should reflect on the content and competencies needed to implement IE by establishing the required actions in terms of preparation (both initial and ongoing), support and resource provision, and quality systems establishment, with special attention being paid to novice teachers and teachers with more years of teaching experience.

## Figures and Tables

**Table 1:** The Evaluation of Teacher Preparation for Inclusion Questionnaire (CEFI-R) dimensions.

Item	Dimension
1. I would prefer to have students with special needs in my classroom.	(1) Diversity conception
2. Students with special needs do not disrupt the classroom routine or their classmates’ learning.	
3. We should place students with special needs in regular schools even if we do not have the appropriate preparation.	
4. Students with special needs can follow the day-to-day curriculum.	
5. I am not concerned about the potential increased workload from the presence of students with special needs in my classroom.	
6. I can teach differently according to my students’ characteristics.	(2) Methodology
7. I can design lessons, bearing the diversity of students’ needs in mind.	
8. I can adapt the evaluation process, bearing the diversity of students’ needs in mind.	
9. I can handle and adapt my teaching materials to respond to my students’ needs.	
10. I can adapt my communication skills to ensure that all students are included in my classroom.	
11. * *Joint teacher-support teacher planning would make it easier to provide support within the classroom.*	(3) Supports
12. The best way to support students is to have the support teacher in the classroom rather than a separate support room.	
13. The role of the support teacher is to work with the whole class.	
14. The place of the support teacher is in the regular classroom with each of the teachers.	
15. An educational project should be reviewed with the different agents of the educational community participation.	(4) Community participation
16. A close relationship between the teaching staff and the rest of the educational stakeholders (parents’ and neighbours’ associations, school councils, etc.) is essential.	
17. Schools must encourage parent and community participation.	
18. * *Each school member (teachers, parents, students, other professionals) is a school key element.*	
19. Schools must work together with neighbourhood resources.	

* Deleted items. Note: The CEFI-R questionnaire was created in Spanish. These items have been translated into English to facilitate reading, and a cross-cultural adaptation into English has not been performed.

**Table 2:** Sample characterization for this study (*n* = 1275).

Variable	Categories	*N*	%
Sex	Men	350	27.5
	Women	925	72.5
Age (years)	Under 30	130	10.2
	Between 31 and 40	385	30.2
	Between 41 and 50	428	33.6
	Over 50	332	26
Educational stage	Early education	221	17.3
	Primary education	605	47.5
	Secondary education	449	35.2

*N*: number; %: percentage.

**Table 3:** Frequencies and percentages of teachers’ answers to the three dichotomous questions.

Questions		Answers	
		Yes	No
(1) Do you think that you were adequately prepared by your initial preparation to respond to the diversity of your students’ needs?	*N* (%)	336 (26.4)	939 (73.6)
(2) Has ongoing preparation helped you to respond to the diversity of your students’ needs?	*N* (%)	985 (77.3)	290 (22.7)
(3) Would you be willing to attend courses on IE?	*N* (%)	1146 (89.9)	129 (10.1)

*N*: number; %: percentage.

**Table 4:** Correlations between The Evaluation of Teacher Preparation for Inclusion Questionnaire (CEFI-R) dimensions and the teachers’ years of teaching experience.

Dimensions	Years of Experience *ρ* (*p*)
(1) Diversity conception	−0.07 (0.006 **)
(2) Methodology	0.02 (0.466)
(3) Supports	−0.14 (<0.001 **)
(4) Community participation	−0.15 (<0.001 **)

Correlation is considered significant at ** *p* < 0.01. Each score is based on a Likert scale (1–4): 1 being “Strongly Disagree”; 2, “Partially Disagree”; 3, “Partially Agree”; and 4, “Strongly Agree”.

**Table 5:** Correlations between The Evaluation of Teacher Preparation for Inclusion Questionnaire (CEFI-R) dimensions and the teachers’ age group.

Dimensions	Age *ρ* (*p*)
(1) Diversity conception	−0.11 (<0.001 **)
(2) Methodology	−0.03 (0.270)
(3) Supports	−0.13 (<0.001 **)
(4) Community participation	−0.16 (<0.001 **)

Correlation is significant at the ** *p* < 0.01; Each score is based on a Likert scale (1–4): 1 being “Strongly Disagree”; 2, “Partially Disagree”; 3, “Partially Agree”; and 4, “Strongly Agree”.

**Table 6:** Reliability values from The Evaluation of Teacher Preparation for Inclusion Questionnaire (CEFI-R) dimensions through Cronbach’s alpha coefficient.

Dimensions	Cronbach’s Alpha
(1) Diversity conception	0.79
(2) Methodology	0.93
(3) Supports	0.77
(4) Community participation	0.94

Values are considered acceptable between 0.60 and 0.70 and excellent between 0.70 and 0.90 [46].

## Data Availability

The datasets used during the current study are available from the corresponding author upon reasonable request.

## Appendix A

The Evaluation of Teacher Preparation for Inclusion Questionnaire is the translated version for the original Cuestionario para la Evaluación de la Preparación del Profesorado para la Inclusión (CEFI-R), created in Spanish.

1 Preferiría no tener en mi aula alumnos con necesidades específicas de apoyo educativo
2 Un niño con necesidades específicas de apoyo educativo interrumpe la rutina del aula y perjudica el aprendizaje de sus compañeros
3 No debemos escolarizar alumnos con necesidades educativas especiales en centros ordinarios hasta que no tengamos la formación adecuada para ello
4 Los alumnos con necesidad específica de apoyo educativo no pueden seguir el día a día del curriculum
5 Me preocupa que mi carga de trabajo se incremente si tengo alumnos con necesidades específicas de apoyo educativo en mi clase
6 Sé cómo enseñar a cada uno de mis alumnos de manera diferente en función de sus características individuales
7 Sé cómo elaborar las unidades didácticas y las clases teniendo presente la diversidad de los estudiantes
8 Sé cómo adaptar mi forma de evaluar a las necesidades individuales de cada uno de mis alumnos
9 Sé cómo manejar y adaptar los materiales didácticos para responder a las necesidades de cada uno de mis alumnos
10 Soy capaz de adaptar mis técnicas de comunicación para asegurarme de que todos los alumnos puedan ser incluidos con éxito en el aula ordinaria
11 *La planificación conjunta profesor-profesor de apoyo facilitaría que los apoyos se proporcionaran dentro del aula*
12 Creo que la mejor manera de proporcionar apoyo a los alumnos es que el profesor de apoyo se incorpore al aula, en lugar de hacerlo en el aula de apoyo
13 La función del profesor de apoyo es trabajar con todo el alumnado de mi aula
14 Considero que el lugar del profesor de apoyo está dentro del aula ordinaria con cada uno de los profesores
15 El proyecto educativo debería revisarse con la participación de los distintos agentes de la comunidad educativa (profesores, padres, alumnos…)
16 Es fundamental que haya una relación muy estrecha entre el profesorado y el resto de agentes educativos (AMPA, asociación de vecinos, consejo escolar…)
17 La escuela debe fomentar la implicación de los padres y de la comunidad
18 *Cada miembro del centro educativo (profesores, padres, alumnos, otros profesionales) es un elemento fundamental del mismo*
19 El centro debe trabajar de forma conjunta con los recursos del barrio (biblioteca, servicios sociales, servicios sanitarios…)
